# Feasibility of a Customized, In-Home, Game-Based Stroke Exercise Program Using the Microsoft Kinect® Sensor

**DOI:** 10.5195/ijt.2015.6177

**Published:** 2015-11-20

**Authors:** RACHEL PROFFITT, BELINDA LANGE

**Affiliations:** 1 MRS. T.H. CHAN DIVISION OF OCCUPATIONAL SCIENCE & OCCUPATIONAL THERAPY, UNIVERSITY OF SOUTHERN CALIFORNIA, LOS ANGELES, CA, USA; 2 INSTITUTE FOR CREATIVE TECHNOLOGIES, UNIVERSITY OF SOUTHERN CALIFORNIA, PLAYA VISTA, CA, USA

**Keywords:** Home exercise program, motivation, stroke, virtual reality

## Abstract

The objective of this study was to determine the feasibility of a 6-week, game-based, in-home telerehabilitation exercise program using the Microsoft Kinect® for individuals with chronic stroke. Four participants with chronic stroke completed the intervention based on games designed with the customized Mystic Isle software. The games were tailored to each participant’s specific rehabilitation needs to facilitate the attainment of individualized goals determined through the Canadian Occupational Performance Measure. Likert scale questionnaires assessed the feasibility and utility of the game-based intervention. Supplementary clinical outcome data were collected. All participants played the games with moderately high enjoyment. Participant feedback helped identify barriers to use (especially, limited free time) and possible improvements. An in-home, customized, virtual reality game intervention to provide rehabilitative exercises for persons with chronic stroke is practicable. However, future studies are necessary to determine the intervention’s impact on participant function, activity, and involvement.

Stroke is the leading cause of serious, long-term disability in the United States. Every year, approximately 800,000 people experience a new or recurrent stroke (Go et al., 2013). After an initial stroke, varying degrees of spontaneous recovery can occur; some survivors are able to return to their usual activities of daily living (ADLs) (Hartman-Maeir et al., 2007). Typically, six months after the initial event, a large percentage of individuals experience deficits including hemiparesis (50%) and dependence in ADLs (26%) (Go et al., 2013). In these cases, inpatient rehabilitation programs are the primary means to address and improve impaired physiological, motor, and cognitive functioning (Woodson, 2008). Unfortunately, for those with residual deficits in functioning, only 31% reported receiving outpatient rehabilitation (Centers for Disease Control, 2007).

In-home exercise programs are a viable option for stroke survivors who require continued rehabilitation to generate improvements in function; for those who require regular exercise and daily physical activity to maintain gains and prevent declines after outpatient rehabilitation; and for patients whose insurance coverage for structured rehabilitation has ended. These programs typically consist of a list of exercises with illustrated instructions that patients must read, understand, and implement on their own. Adherence to these self-guided programs is low because factors including fatigue, poor health, lack of motivation, and musculoskeletal issues often prevent patients from either initiating or maintaining the exercise programs (Jurkiewicz et al., 2011; Payne et al., 2001; Shaughnessy et al., 2006). Further, among those who implement self-guided programs, adherence is often very difficult to quantify or assess because typically patients do not consistently or accurately record their progress.

To mitigate barriers to implementing in-home exercise programs, it is necessary to provide patients with tools that motivate them to practice exercise activities in a safe, structured, and easily monitored manner. A novel way to achieve this in the area of stroke rehabilitation is through the use of interactive technologies such as video games and virtual reality (VR). The use of interactive technologies and VR for exercise and rehabilitation has expanded rapidly over the past 15 years. Systematic reviews and clinical trial data suggest that VR technology can be used to improve motor skill rehabilitation for a range of functional deficits (Adamovich et al., 2005; Henderson et al., 2007; Merians et al., 2002; Stewart et al., 2007). However, the technologies and systems described in these reviews are limited because they are expensive, not portable into the home setting, or use outdated technology and software.

To address these limitations, we have developed a VR rehabilitation software called Mystic Isle that combines the customized nature of rehabilitation with components of self-determination theory (Ryan & Deci, 2000; Sheldon & Filak, 2008) and game engagement (Przybylski et al., 2010). This highly specialized rehabilitation software, in combination with a high-fidelity movement sensor, the Microsoft Kinect® camera, provides the following benefits that are superior to those obtained through other currently available VR systems for rehabilitation: (1) targeted improvements in physiological, motor, and/or cognitive performance; (2) customization to patient treatment goals, preferences, and need; (3) individualized therapy without requiring an intensive one-on-one time commitment by a therapist; (4) easily transportable into the home, providing an expedient and practical mode of ongoing care; (5) immediate feedback to the patient; and (6) a record of quantitative performance data easily accessed by the therapist. Further, Mystic Isle allows therapists with little to no programming skills to modify the delivery parameters for participant interaction across a variety of relevant dimensions and to easily extract and view performance data (Lange et al., 2011; Lange et al., 2012). The primary purpose of this study was to explore the feasibility and utility of Mystic Isle as a 6-week, game-based, in-home exercise program for individuals with chronic stroke. A secondary purpose was to provide a preliminary assessment of the impact of game involvement on clinical outcomes.

The Institutional Review Board of the University of Southern California approved this study.

## METHODS

### STUDY PARTICIPANTS

Four individuals with chronic stroke participated in this feasibility study. Participants were chosen based on the following criteria: (1) had sustained a stroke ≥ 6 months prior to study commencement; (2) ability to read English at a 6^th^ grade level; and (3) possess in-home internet connection. Study candidates were excluded if medical contraindications (e.g., seizure disorders) prevented them from playing video games. Table 1 depicts the participants’ demographic and clinical characteristics.

Participant 1 had left hemiparesis in both the arm (moderate to severe upper extremity motor function deficits) and leg that manifested as mild to moderate mobility impairments, mild impact on ADL performance, and minimal impact on instrumental activities of daily living (IADL) performance. Participant 4 had a similar clinical presentation; however, he reported that his hemiparesis had a mild impact on his performance of IADLs. With hemiparesis on her right side (mild deficits in upper extremity motor function), Participant 2 reported a mild impact on her mobility and negligible impact on her performance of ADLs and IADLs. None of these participants had detectable cognitive deficits. In contrast, Participant 3 had no motor deficits due to stroke, but demonstrated expressive aphasia and cognitive deficits that moderately impacted her performance of IADLs

### INTERVENTION

#### MYSTIC ISLE GAME

**Figure 1:**
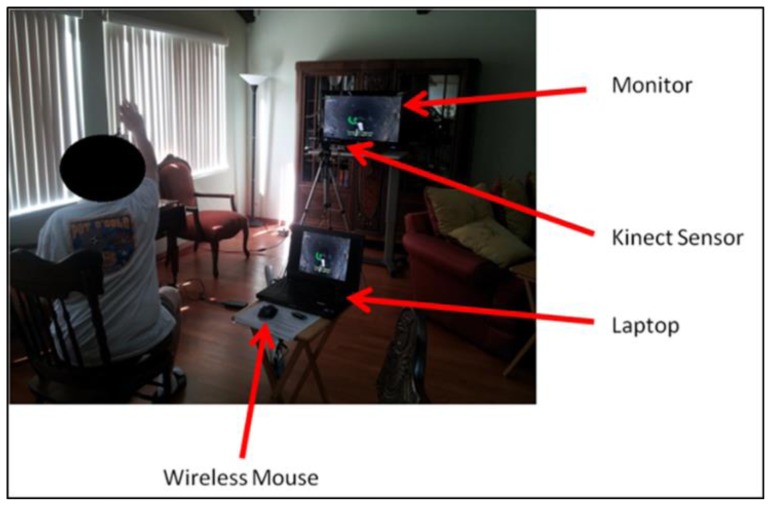
Mystic Isle game set-up in a participant’s home. The participant is seated and reaching forward with his right arm. Visible are the monitor, the Kinect sensor, the laptop, and the wireless mouse.

The Mystic Isle software (Lange et al., 2012) runs on a standard desktop or laptop PC. The Microsoft Kinect® camera connects to the computer via USB and serves as the input sensor that detects and tracks a player’s joint movements in 3D space (Figure 1). The software and components are designed to be easy to assemble and navigate.

Designed for use by an occupational therapist (OT), the software contains a control panel through which the game can be calibrated and modified based on the player’s physical rehabilitation and exercise treatment plan needs and goals. For example, it allows for tailoring of parameters such as game timing (for rapid game start), physical tasks, visual preferences, repetition number, and challenge level. One additional feature is that the preferences and calibrations can be updated as needed throughout the course of the intervention.

#### GAME SELECTION AND CALIBRATION

An OT administered the Canadian Occupational Performance Measure (COPM) (Law, 1998) to identify each participant’s occupational performance problems and generated five treatment goals. The OT subsequently customized the game and calibrations in accord with the assessment findings (Table 2). For example, Participant 2 identified a goal of being able to kneel and reach forward to clean her bathtub. In the custom calibration, participant 2 kneeled on the ground and reached forward for virtual objects during game interaction. This challenged and helped restore the specific abilities necessary to complete the functional task of cleaning the bathtub: trunk control, core strength, and reaching. Each week the OT reviewed the adequacy of the game settings and calibrations in relation to automated progress report findings. When needed, she made changes to game settings and calibrations based on her clinical judgment and on participant progress by remotely logging-in to the participant’s home-based computer.

#### IN-HOME SETUP

Two researchers and the OT visited each participant’s home to assist in setting up the Mystic Isle software, monitors, Microsoft Kinect® sensor, and computer peripherals. The in-home setup location was determined in cooperation with each participant and, if necessary, his/her family members/caregivers. The location was required to: (1) provide adequate space for game equipment and movement and (2) allow for Internet connectivity or wireless Internet access. In addition to instructing participants in how to set up the technology, researchers also taught them how to troubleshoot technical issues, navigate the gaming menu, and load intervention tasks.

#### INTERVENTION DURATION

Each participant was instructed to achieve at least 4 hours of game play per week over the 6-week intervention period. The duration of each game play session was left to the participant’s discretion. For this reason, game play session duration varied among participants. None of the study participants were receiving therapy outside of this study.

## OUTCOMES

### FEASIBILITY AND USABILITY OF THE MYSTIC ISLE GAME (PRIMARY OUTCOMES)

The primary outcomes of this study were the usability and feasibility of the Mystic Isle in-home exercise program for persons with chronic stroke. These outcomes were assessed at post-intervention only using an embedded design, mixed-methods approach (Creswell, Klassen, Plano Clark, & Smith, 2011). Feasibility and usability were quantitatively measured using the Game Experience Questionnaire, derived from IBM measures of system usability (Lewis, 1995), and the System Usability Questionnaire (Bangor, Kortum, & Miller, 2009). Both instruments utilize a 5-point Likert scale rating. Qualitative assessment of feasibility and usability was determined via a semi-structured interview that focused on the following: (1) the attractiveness and appeal of the Mystic Isle intervention; (2) system integration, including technology set-up, into the home; and (3) the rehabilitative potential of Mystic Isle. Additionally, the Mystic Isle software provided data on the exact number of minutes each participant spent playing the game each day.

### CLINICAL IMPACT (SECONDARY OUTCOMES)

Upper extremity function, perceived activity performance, balance confidence, and quality of life were assessed at baseline, as well as 2 weeks later (i.e., just prior to intervention commencement, and following intervention). Upper extremity function was assessed with the Fugl-Meyer Upper Extremity Assessment (Fugl-Meyer, Jassko, Leyman, Olsson, & Stenlind, 1975). This performance-based assessment is well validated and reliable for the chronic stroke population (Duncan, Propst, & Nelson, 1983; Hsieh et al., 2009). Perceived activity performance was assessed with the Canadian Occupational Performance Measure (COPM) (Law, 1998). Structured as an interview and self-rating of performance and satisfaction with performance, the COPM has high validity and reliability for persons with chronic stroke (Cup, Scholte op Reimer, Thijssen, & van Kuyk-Minis, 2003). Balance confidence was assessed through a self-report measure, the Activities-Specific Balance Confidence Scale (Botner, Miller, & Eng, 2005). An additional self-report measure, the Stroke-Specific Quality of Life Scale (Williams, Weinberger, Harris, Clark, & Biller, 1999), was utilized to assess stroke-related quality of life. Both the Activities-specific Balance Confidence Scale and the Stroke-Specific Quality of Life Scale have good validity and reliability for the chronic stroke population (Botner, Miller, & Eng, 2005; Lin et al., 2010).

### STATISTICAL ANALYSIS

The results of the Game Experience Questionnaire and System Usability Scale were expressed as a distribution of Likert scale responses for each participant. The semi-structured interview data were analyzed using a grounded theory approach (Corbin & Strauss, 2008). Two independent researchers coded the data to detect emerging themes. They also met frequently for inter-coder agreement checks. The quantitative data were then synthesized with the qualitative data to substantiate emerging themes. For the clinical evaluation data, baseline and pre-intervention scores for each measure were averaged to produce a pre-intervention score for each study participant. The differences in the participants’ post- and pre-intervention scores were compared to the Minimal Detectable Change (MDC) score for each clinical assessment tool. The MDC was used for this study because it provides a criterion for the smallest amount of change in an outcome measure that corresponds to perceptible change in ability or functional status.

## RESULTS

### PARTICIPANT EXPERIENCE AND SYSTEM USABILITY

Three out of four participants rated the system as “usable” or “very usable.” This was evidenced by their average ratings of 1.46, 2.19, and 2.76 on the Game Experience Questionnaire and System Usability Scale, where 1 = very usable, and 5 = not usable at all. Participant 3 had an average rating of 4.42 due to reported difficulty understanding the directions to start the game on the laptop. Despite the therapist and study team’s best efforts to include visual cues and reminders, Participant 3 experienced frustration loading the game and navigating through the three steps required to begin playing. Moreover, Participant 3 also continued to attempt playing the game without a caregiver present to load it or to troubleshoot, even though the therapist and study team recommended she include a caregiver in the process. Participant 4 experienced minor technical issues but was able to resolve them during a phone call to the treating therapist or study team. Once the intervention was completed, participants identified factors that facilitated the use of Mystic Isle as an in-home intervention, identified barriers, and made suggestions for future software improvements.

### FACTORS THAT FACILITATED USE

Participants indicated they liked the customized aspect of the Mystic Isle. They claimed they were inspired to play and enjoyed the games because they were tailored to help them achieve their particular goals. As one participant stated, “It was fun when I could tailor a game to me.” The overall desire to improve and “get [their] bod[ies] and mind[s] better” was the main incentive for all participants. Further, they indicated that they appreciated having a variety of games to help them reach their goals and multiple means to improvement: “I enjoyed that they [Mystic Isle games] were [each] different.” Furthermore, the three participants without cognitive difficulties were additionally motivated by the motor + cognitive challenge games.

### BARRIERS

Time management was the main barrier identified by participants. On average, participants played 30 minutes per day, 5 days per week; totaling 2.5 hours each week. Participant 2 averaged 3.5 hours/week, whereas Participant 3 only averaged 1 hour/week. All participants found it difficult to follow the 4-hour recommended length of game-playing each week due to busy schedules or previous commitments. For example, all participants were regularly engaged in activities in and outside of the home such as yoga, book club, and stroke support groups. In the words of one participant: “I was just trying to figure out when to get it in because I am so busy.” Additionally, participants reported that they were often fatigued or stressed after a full day of activity: “I just feel so overwhelmed sometimes, [with] all the things I got to do.” To overcome this barrier, some of the participants attempted to play in the morning before going out or divided the time invested into multiple short durations throughout the day. Seventy-five percent of game play sessions across participants occurred exclusively in the afternoon or evening.

For Participant 3, accessing the game on the computer (e.g., finding the icon to click, following the steps to log-in) presented a barrier to play. Participant 3 was able to play the game with verbal cues when the OT was present in the home; however at other times, she did not use assistance from caregivers to load or play the game. Due to her cognitive deficits, she had difficulty processing and following directions, both written and pictorial. However, none of the participants mentioned the physical environment or physical technology set-up as a barrier to use.

### SUGGESTIONS FOR FUTURE USE

Participants provided the following feedback on how to improve the Mystic Isle intervention: (1) Increasing the amount of choice on the part of the player; (2) Including background music; (3) Providing more on-screen performance feedback; (4) Maintaining the on-screen instructions, but allowing the player to progress in the game without having to read them; and (5) Adding an on-screen “Help” link for technical issues and troubleshooting.

### CLINICAL EVALUATION

Participants 2 and 4 reported an increased ability in the self-care domain on the Stroke-Specific Quality of Life scale (post- and pre-intervention differences of 4.0 and 9.0, respectively), which was greater than the MDC of 4.0. Participant 2 obtained an increased score in COPM satisfaction (a post- and pre-intervention difference of 2.4), which was greater than the MDC of 1.75. No scores on other clinical evaluation measures were greater than the MDC for each respective measure (Table 3).

## DISCUSSION

The primary purpose of this study was to explore the feasibility and utility of a 6-week, game-based, in-home exercise program for persons with chronic stroke. Three of the four participants were able to successfully use the Mystic Isle intervention as evidenced by their ratings on usability scales and their subjective responses to open-ended interview questions. However, the hours invested by all participants fell short of the recommended time. Participant 2 consistently completed the intervention for the most amount of time per week (3.5 hours/week for all 6 weeks). She also experienced improvements in clinical evaluation measures outside of the MDC, but these improvements were subjectively self-reported. Although Participant 2 had higher baseline levels of function, the two participants with greater deficits in motor and ADL function were still able to use the Mystic Isle game in their home. The majority of recent studies investigating interventions for the chronic stroke population target persons much like Participant 2 who have higher levels of motor function (i.e., some hand movement and a score greater than 45 on the Fugl-Meyer Assessment-Upper Extremity) (Combs, Kelly, Barton, Ivaska, & Nowak, 2010; Egan, Kessler, Laporte, Metcalfe, & Carter, 2007; Pang, Eng, Dawson, McKay, & Harris, 2005). The results of this study suggest that it may be feasible to implement an in-home intervention with stroke survivors with lower levels of motor and daily function.

Even with lower levels of motor and daily functioning, the participants were able to use the in-home technological intervention; however, poorer cognitive ability impeded game engagement. As mentioned above, despite the OT’s efforts to remotely troubleshoot and provide reminders, cognitive deficits were found to be a significant barrier to Participant 3’s engagement in the game. It is unclear whether ongoing caregiver support would have lessened or even eliminated this problem. This finding suggests that as it stands, the Mystic Isle game may be most appropriate for individuals who possess minimal cognitive deficits. In a future study, it may be helpful to determine minimum cut-off scores on the Mini Mental State Examination that are predictive of successful adoption of the intervention.

Even in the absence of cognitive deficits, the technology itself in some instances proved difficult to use. For example, Participant 4, who suffered no cognitive deficits, had issues starting and playing Mystic Isle including logging into the game and selecting the appropriate game tasks. Consequently, he had to intermittently stop playing the game to seek help from the study team, inadvertently impacting the frequency of use and revealing limitations of using Mystic Isle as a self-directed intervention. Similarly, in one of our recent studies involving older adults aged 65 or older, we found that Mystic Isle was described as lacking sufficient user-friendliness. The older adults in that study indicated they would be more likely to adopt the game-based intervention if the system was simpler, more intuitive, and had an easy “push-button-start” (Proffitt & Lange, 2013). Currently, our investigative team is modifying the Mystic Isle game based on this feedback.

Although cognitive deficits and technological concerns impeded use in this study, the primary reason all study participants did not achieve the 4-hour per week intervention goal was due to the additional activities in which they were engaged. As stated earlier, participants claimed that they could not devote sufficient time to the exercise program because they were either too busy or simply overwhelmed with all of the things they had to do. Some evidence suggests that, even though many persons with chronic stroke report returning to many of their pre-stroke activities, the amount of time and effort required to complete these activities often increases, even dramatically, in comparison to pre-stroke levels (Mayo, Wood-Dauphinee, Cote, Durcan, & Carlton, 2002). For many stroke survivors, the motivation to complete exercises and therapeutic interventions is strong, but having sufficient time to do so can be problematic (Proffitt & Lange, 2013). Consequently, tailoring game sessions and exercises so that they can be completed with minimal effort and in short amounts of time is likely to facilitate adoption on an ongoing basis by stroke survivors.

Many studies have revealed that adherence in self-directed programs tends to be poor (Chen, Neufeld, Feely, & Skinner, 1999; Forkan et al., 2006; Jurkiewicz, Marzolini, & Oh, 2011). The studies by Forkan and colleagues (2006) and Chen and colleagues (1999) utilized a common method of tracking home exercise program adherence: an exercise log or report completed by the patient. Jurkiewicz and colleagues (2011) did not report specific methods for tracking adherence; however they provided suggestions to increase adherence including increasing support from family members and physicians. Suggestions provided by Chen and colleagues (1999) included a link to the health belief model by suggesting that therapists ensure that patients feel confident in their abilities to complete the prescribed exercises. This is linked to one of the central tenets of Self- Determination Theory that underlies Mystic Isle: competence. Additionally, Forkan and colleagues (2006) reported that the largest barrier to adherence in their study was change in health status, and the authors recommended return visits to the clinic to update the program. The advantage of using Microsoft Kinect® sensor technology in the home is that the OT (or any other care provider) can remotely check progress and update the program without requiring the patient to spend precious time in travel to the clinic. Further, Mystic Isle provides an objective measure of the amount of time a user plays a game, the number of repetitions a player achieves, along with other valid kinematic data (Fern’ndez-Baena, Susin, & Lligadas, 2012) on performance. Not only are such reports helpful for treating therapists, they also create an incentive for persons with stroke to adhere to a program and improve on performance. In this regard, participants indicated that the combination of the customized nature of the games, the relationship of the exercises to self-identified goals, and the objective technologically-provided feedback were key to motivating them to stick to the program.

## LIMITATIONS

The small sample size of this study limits the generalizability of the findings. A larger pilot study is necessary to determine the appropriate sample size for a randomized trial aimed at assessing the efficacy of the Mystic Isle in-home intervention in improving motor and ADL function in persons with chronic stroke. Additionally, future studies are necessary to determine the optimal dose and length of the intervention. The limitations of this study were that participants were not held to a consistently scheduled protocol, and the length of the intervention was only 6 weeks. Duncan and colleagues (2005) provide some parameters in their guidelines for home-based and community-based stroke rehabilitation. Recent studies exploring the feasibility and assessing the efficacy of commercial video games and customized video games for persons with chronic stroke are detailed in a meta-analysis and have provided further parameters for the appropriate dosage (Saposnik & Levin, 2011). We are currently designing the parameters for a larger, pilot study using Mystic Isle as the intervention.

## CONCLUSION

Home exercise programs are an excellent and cost-effective means for stroke survivors to incorporate activity and exercise into their daily lives. We explored the feasibility of using a customized, in-home, game-based intervention called Mystic Isle for persons with chronic stroke. This study demonstrated that Mystic Isle is a feasible in-home exercise option for at least some persons with chronic stroke. Including the customized exercises by the OT in combination with remote monitoring provided the participants with an enjoyable and motivating intervention. The main barrier to use was the limited amount of time that the participants had to devote to completing the intervention. Additionally, Mystic Isle may not be appropriate for individuals with cognitive deficits unless they are provided with close supervision by a caregiver. In order to determine the optimal target population that would benefit from Mystic Isle in improving motor function or performance of ADLs, larger trials must be conducted.

## Figures and Tables

**Table 1:** Characteristics of the Participants

Characteristics	Participant			

	1	2	3	4
	
Gender	M	F	F	M
Age (years)	55	54	64	56
Side of Paresis	L	R	N/A	L
Because of your stroke, do you have any difficulty with:				
Mobility, strength, and fine motor skills (X/36)*	13	6	2	13
Activities of Daily Living (ADLs) (X/21)*	7	0	0	4
Instrumental Activities of Daily Living (IADLs) (X/18)*	4	1	8	3
Fugl-Meyer Assessment-Upper Extremity	24	45	66	25

Note.

* Higher score = greater difficulty

**Table 2:** Canadian Occupational Performance Measure (COPM) Goals and Associated Game Sessions for Each Participant

Participant	COPM Goal	Game Session Exercises
1	Walk on uneven ground	Step up onto platform Reach with right arm Reach with both arms + leg extension exercises
	Fold laundry	Reach with left arm Reach with both arms while standing and then sitting
	Fasten zippers	Reach with left arm while standing
	Find items in a crowded environment	Play memory games during sitting and standing tasks
	Read faster	Perform matching games while sitting and standing tasks

2	Chop food	Reach with right arm
	Kneel to clean bathtub	Reach with left arm in tall kneeling Squat Sit-to-stand
	Use railing while going up/down stairs	Step forward Reach in standing position
	Remember names	Play Memory games while stepping forward
	Put hair in a ponytail	High reach with right arm

3	Reading	Remember words Choose antonym
	See items on left side of visual field	Dual-task game Sort objects
	Communicate with others	Recall words
	Manage money during transactions	Add two numbers Match numbers Remember numbers
	Maintain focus for extended periods of time	Dual-task game for extended period of time (session duration > 10 minutes)

4	Fold clothes	Reach with left and right arm in standing
	Walk for longer periods of time	Reach both arms in standing for extended period of time (session duration > 5 minutes) Reaching with both arms while standing on compliant surface
	Walk on sand at beach	Reach alternately with left and right arms while standing on compliant surface
	Stabilize items using impaired side	Reach with left arm
	Button pants	Reach with left arm

**Table 3:** Results of Clinical Outcome Measures for Each Participant at Three Time Points

Clinical Outcome Measure	Baseline				Pre				Post			
	1	2	3	4	1	2	3	4	1	2	3	4
Fugl-Meyer Assessment-Upper Extremity (x/66)	24	45	66	25	24	44	66	28	24	42	66	33
Canadian Occupational Performance Measure												
Performance (x/10)	3.6	2.4	3.4	1.4	3.4	2.6	3.4	1.2	4.2	3.6	1.2	2
Satisfaction (x/10)	1	2	1.8	1	2.2	1.8	1.4	1	2.4	2.8	4	2.2
Activities-specific Balance Confidence Scale (%)	59.38	75.31	88.75	59.69	70.63	74.25	81.25	65.63	71.88	78.75	94.38	54.38
Stroke-specific Quality of Life Scale												
Energy (x/15)	7	15	6	13	6	15	11	14	8	15	15	11
Family Roles (x/15)	9	12	5	11	13	12	8	10	12	15	9	11
Language (x/25)	20	25	5	25	24	25	10	23	6	25	15	25
Mobility (x/30)	25	30	29	27	14	30	28	20	18	30	30	23
Mood (x/25)	13	24	13	24	20	23	9	21	22	25	16	21
Personality (x/15)	5	12	6	12	7	9	6	13	6	13	7	10
Self-Care (x/25)	12	24	23	24.5	14	24	19	21	22	22	25	21
Social Roles (x/25)	17	16	5	18	8	10	10	12	10	20	17	15
Thinking (x/15)	8	15	3	9	6	15	6	9	8	15	3	10
Upper Extremity Function (x/25)	12	23	20	13	14	20	20	9	14	24	21	11
Vision (x/15)	13	15	13	15	12	15	11	15	14	15	13	15
Work/Productivity (x/15)	14	8	9	8	8	13	9	9	6	9	9	10
Total (x/245)	155	219	137	199.5	146	211	147	176	146	228	180	183
